# Impacts of Six Methods of Extraction on Physicochemical Properties, Structural Characteristics and Bioactivities of Polysaccharides from *Pholiota nameko* Residue

**DOI:** 10.3390/foods14173071

**Published:** 2025-08-30

**Authors:** Jingbo Zhang, Yajing Bai, Xiaoxue Zhang, Yiyao Wang, Zongshu Li, Chengguang He, Lili Guan

**Affiliations:** 1 College of Life Sciences, Engineering Research Center of Bioreactor and Pharmaceutical Development, Ministry of Education, Jilin Agricultural University, Changchun 130118, China; 18143663850@163.com (J.Z.); 13633166476@163.com (Y.B.); 13394435435@163.com (Y.W.); 15647650966@163.com (Z.L.); 2 College of Animal Science and Technology, Jilin Agricultural University, Changchun 130118, China

**Keywords:** *Pholiota nameko* residue, polysaccharides, extraction methods, function characteristics, bioactivities, immunomodulatory

## Abstract

By integrating waste valorization with green extraction, in the current study, the impacts of distinct extraction methods on the extraction yield, structural characterization, in vitro antioxidant abilities and in vitro immunomodulatory activity of polysaccharides from *Pholiota nameko* residue (PNRP) were determined, providing assistance for the resource utilization of *Pholiota nameko.* Six PNRPs were obtained by hot water extraction, ultrasonic-assisted extraction, acid-assisted extraction, base-assisted extraction, acid–base extraction and hot water–alkaline-assisted extraction, named PNRP-HWE, PNRP-UAE, PNRP-AE, PNRP-BE, PNAP-ABE and PNRP-HAE, respectively. PNRPs were heteropolysaccharides with similar functional groups, abundant branched chains and a triple helix conformation, but varied monosaccharide molar ratios and molecular weights (382.6–601.7 kDa). PNRP-HAE exhibited the highest yield (3.92%) and superior antioxidant activities, including DPPH, ABTS and hydroxyl radical scavenging capacities, attributed to its low molecular weight and high xylose content. Additionally, PNRP-HAE and PNRP-UAE demonstrated potent immunomodulatory effects by enhancing macrophage phagocytosis and cytokine secretion (NO, IL-1β, IL-6, TNF-α). These findings highlight HAE as an optimal method for extracting high-quality PNRPs, offering a sustainable strategy for valorizing mushroom residue in functional foods and nutraceuticals.

## 1. Introduction

With the breakneck growth of the edible mushroom industry, China has become the largest producer of edible mushrooms [1]. A large amount of mushroom residue is turned out in the production of edible mushrooms. In 2020, the amount of mushroom residue produced in China reached 132–203 million tons [2], which has caused a large amount of resource waste and new environmental problems [3]. The arbitrary stacking of mushroom residue can easily cause the growth of fungi and pests, and emit toxic gases, causing serious environmental pollution [4]. Therefore, the recycling and reuse of mushroom residue has become an urgent topic that needs to be addressed at present. Polysaccharides, the primary bioactive components in mushrooms, offer a promising way to valorize the residue. Recovering polysaccharides from waste transforms them into high-value products, creating viable industrial applications [5]. Existing studies have confirmed the diverse biological activities of polysaccharides derived from mushroom residue, which lay a solid foundation for their application. For example, polysaccharides from *Cordyceps militaris* SU-12 residue (RPS) exhibit anti-hyperlipidemic and hepatoprotective effects [6], while those from *Pleurotus eryngii* enzymatic residue (PERP) show anti-aging properties by boosting antioxidant capacity, reducing lipid peroxidation and mitigating multi-organ damage [7]. Our prior work identified a novel polysaccharide from *Flammulina velutipes* residue (FVRP) with strong in vitro antioxidant activity, heavy metal detoxification capabilities and the ability to alleviate Pb-induced kidney injury by regulating the Akt/GSK3β/Nrf-2/HO-1 pathway and gut microbiota [8]. Thus, researching their extraction and pharmacological activities is key to advancing mushroom residue reuse, offering solutions to both resource waste and environmental challenges in the industry.

*Pholiota nameko*, a widely commercialized edible mushroom in China, is rich in protein, amino acids, polysaccharides and various trace elements, with documented benefits for gastrointestinal health and immunity [9]. *Pholiota nameko* polysaccharides exhibited strong bioactivities, including antioxidant, antibacterial, lipid-lowering and antitumor activity and other functions [10,11,12,13]. However, research on *Pholiota nameko* residue (PNR) is limited to an adsorbent and antiseptic [14]. Most notably, there were no reports on the research on the *Pholiota nameko* residue polysaccharides (PNRP). The in vitro biological abilities of polysaccharides are closely interrelated to the physicochemical properties and structures (molecular weight, monosaccharide composition, uronic acid content and spatial configuration), which are impacted by various extraction methods [15,16]. The hot water extraction method (HWE) is popularly utilized owing to the balmy extraction conditions and reduced seriously structural damages to polysaccharides, but with certain shortcomings, including being time-consuming, its high energy demand, mega temperature and low extraction rate [17,18,19]. To explore polysaccharides with diverse structures and activities, physical and chemical methods, including acid extraction (AE), alkali extraction (BE) and ultrasonic-assisted extraction (UAE), are commonly employed, as they target different mechanisms (e.g., cell wall disruption via chemical hydrolysis or physical cavitation) to release intracellular polysaccharides [20]. Notably, acidic and alkaline treatments can break bonds between cell wall proteins and glucans to increase polysaccharide release but may damage glycosidic bonds, reducing yields [21]. In contrast, UAE enhances yields through cavitation and mechanical waves that rupture cell walls, offering advantages like high efficiency and minimal harm to active components [22]. Previous studies highlight method-specific effects: for example, microwave-assisted aqueous two-phase extraction (MA-ATPE) achieved a higher yield of polysaccharides from *Hippophae rhamnoides* than microwave-assisted extraction (MAE), UAE or HWE [23]. Extraction methods altered the properties of *Lonicera japonica* polysaccharides, with UAE-extracted *Lonicera japonica* polysaccharides (UE-LJP) showing the highest yield and water-holding capacity but lowest protein content [24]. Similarly, extraction methods affected the molecular weight of *Clitocybe squamulosa* polysaccharides despite consistent monosaccharide compositions [25]. In addition, acid and base extraction methods (ABE) are also commonly used to extract plant polysaccharides, especially for pectic polysaccharides. However, most studies of acid and base extraction performed their work under hot acid/base conditions, which would accelerate the hydrolysis of the rhamnogalacturonan-I (RG-I) domains of pectic polysaccharides [26]. Hot water utilized extrinsic thermal power to swell cells and to change intracellular and extracellular osmotic pressures, promoting the release and diffusion of polysaccharides from banana flower. Alkaline extraction effectively facilitated the release of insoluble polysaccharides from cell walls and their conversion to soluble polysaccharides by degrading the hydrogen bonds between cellulose and hemicellulose, thus raising the ultimate extraction yield of polysaccharides [27]. Tang et al. suggested that this could be the reason why the highest extraction yield of Banana flower polysaccharide (BFP) was achieved with hot water–alkaline-assisted extraction (HAE) [27].

Hence, the present study compares the physicochemical properties and in vitro biological activities of PNRP extracted using HWE, AE, BE, HBE, ABE and UAE. By investigating how these methods modulate the characteristics of PNRP, we aim to establish a foundation for the exploitation of PNRP and facilitate the sustainable reuse of *Pholiota nameko* residue.

## 2. Materials and Methods

### 2.1. Materials

*Pholiota nameko* residue was provided by Hongshi Forest Farm Cooperative (Huadian, China). Next, 1,1-diphenyl-2-picrylhydrazyl (DPPH) was procured from Yuanye Bio-Technology Co., Ltd. (Shanghai, China), and 2,2′-azino-bis (3-ethylbenzothiazoline-6 sulfonic acid (ABTS)) was procured from Aladdin Biotechnology Co., Ltd. (Shanghai, China). Mannose (Man), glucuronic acid (GlcA), rhamnose (Rha), galacturonic acid (GalA), glucose (Glc), galactose (Gal), xyl (Xyl), arabinose (Ara) and fucose (Fuc) were used as standards for the monosaccharide composition assay and supported by Sigma-Aldrich (Shanghai, China) Trading Co., Ltd. (Shanghai, China). Dextran standards with different molecular weights (12.6 63.3 102 302 and 633 kDa) were purchased from Molecular Weight Standard from NIM. The murine macrophage RAW264.7 cells were obtained from Meilune Biotechnology Co., Ltd. (Dalian, China). The nitric oxide (NO) and BCA protein assay kit was purchased from Abbkine Biotechnology Co., Ltd. (Wuhan, China), and cell counting kit-8 (CCK-8) assay kits were purchased from Beyotime Biotechnology (Shanghai, China). ELISA kits for IL-1β, IL-6 and TNF-α were obtained from Bioswamp Life Science Lab (Wuhan, China). Lentinan was obtained from Aladdin Chemical Co., Ltd. (Shanghai, China). Lipopolysaccharide (LPS) was obtained from Sigma-Aldrich Chemical Co., Ltd. (St. Louis, MO, USA). All other drugs and reagents were of analytical grade.

### 2.2. Extraction Procedure

**Figure 1:**
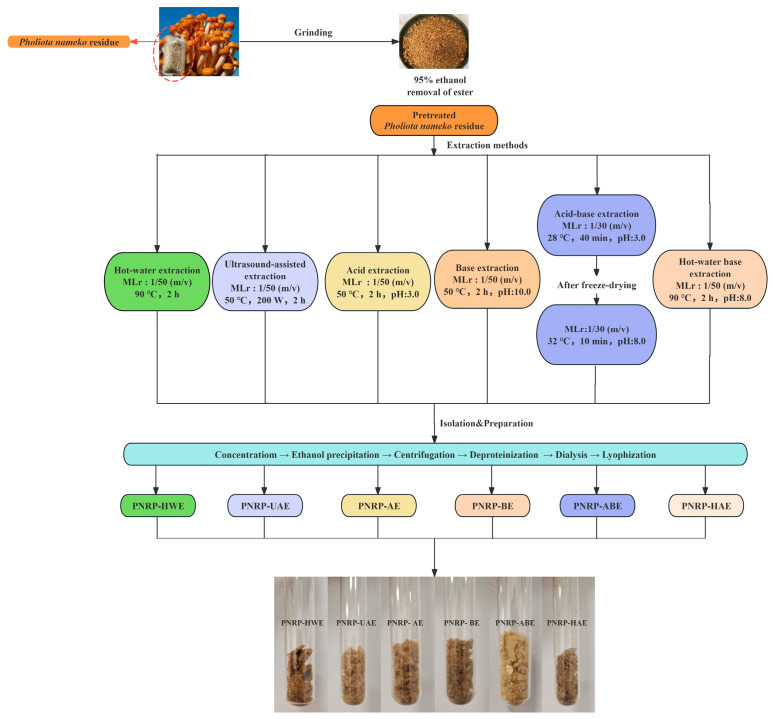
The preparation process of PNRPs. MLr: Material-to-Liquid ratio.

In the current work, six different methods were applied to extract *Pholiota nameko* residue polysaccharides (PNRPs). The detailed extraction process is listed in Figure 1.

#### 2.2.1. Hot Water Extraction (HWE)

*Pholiota nameko* residue powder was soaked with deionized water in a solid–liquid ratio of 1:50 (*w*/*v*) at 90 °C for 2 h [19]. The supernatant was obtained by centrifugation (6000 rpm 10 min) and evaporated at 45 °C, and then after being stirred thoroughly, it was precipitated with 95% ethanol at a final concentration of 3:16 overnight, with the alcohol precipitation carried out in a 4 °C refrigerator for 20 h. The precipitate was deproteinated by the Sevag reagent, and dialyzed with running water for 2 days and deionized water for 2 days (with a membrane cutoff of 7–14 kDa), and then freeze-dried to obtain the *Pholiota nameko* residue polysaccharide (PNRP-HWE).

#### 2.2.2. Ultrasonic-Assisted Extraction (UAE)

The powdered *Pholiota nameko* residue was soaked with deionized water in a ratio of 1:50 (*w*/*v*) under an ultrasonic power of 200 W at 50 °C for 2 h in an Ultrasonic cleaning machine KQ-800KDE (Kun Shan Ultrasonic Instruments Co., Ltd., Kunshan, China) [28]. The following steps were conducted according to HWE procedures to obtain *Pholiota nameko* residue polysaccharides (PNRP-UAE).

#### 2.2.3. Acid-Assisted Extraction (AE)

*Pholiota nameko* residue powder was mixed with 0.05 M HCl solution (pH 3.0) in a solid–liquid ratio of 1:50 (*w*/*v*) and stirred continuously at 50 °C for 2 h [29]. The supernatant was subjected to the same treatment after centrifugation (6000 rpm 10 min) as HWE to obtain the crude *Pholiota nameko* residue polysaccharide (PNRP-AE). The following steps were conducted according to HWE procedures to obtain *Pholiota nameko* residue polysaccharides (PNRP-AE).

#### 2.2.4. Base-Assisted Extraction (BE)

The powdered *Pholiota nameko* residue was soaked with 0.1 M NaOH (pH 10.0) solution in a ratio of 1:50 (*w*/*v*) at 50 °C for 2 h [30]. The following steps were conducted according to HWE procedures to obtain *Pholiota nameko* residue polysaccharides (PNRP-BE).

#### 2.2.5. Acid–Base Extraction (ABE)

The powdered *Pholiota nameko* residue was soaked in 0.05 M HCl solution (pH 3.0) with a ratio of 1:30 (*w*/*v*) at 28 °C for 40 min. After being deproteinated, dialyzed and lyophilization, the crude polysaccharide powder was extracted again with 0.1 M NaOH solution (pH 8.0) at 32 °C and stirred for 10 min according to the previous method [31]. The following steps were conducted according to HWE procedures to obtain *Pholiota nameko* residue polysaccharides (PNRP-ABE).

#### 2.2.6. Hot Water–Alkaline-Assisted Extraction (HAE)

The powdered *Pholiota nameko* residue was soaked with 0.05 M NaOH solution (pH 8.0) in a ratio of 1:50 (*w*/*v*) at 90 °C for 2 h [27]. The following steps were conducted according to HWE procedures to obtain *Pholiota nameko* residue polysaccharides (PNRP-HAE).

#### 2.2.7. The Extraction Yield of PNRPs

The weight of PNRPs’ powder was determined to calculate the extraction yield of PNRPs calculated according to the following formula:

(1) $$\mathrm{Extraction}\;\mathrm{yield}\;(\%)=\frac{mPNRPs}{mPNR}\times 100\%$$

wherein mPNRPs and mPNR are the weight of PNRPs (g) and *Pholiota nameko* residue (g), respectively.

### 2.3. Physicochemical Properties and Structure Characterization

#### 2.3.1. Physicochemical Properties’ Analysis of PNRPs

The contents of polysaccharides, protein, uronic acid and total phenols of PNRPs were detected with the phenol sulfuric acid method [32], Coomassie brilliant blue method [31], sulfuric acid meta hydroxybiphenyl method [33] and folin ciocalteu method [34].

#### 2.3.2. Monosaccharide Composition Determination of PNRPs

The monosaccharide composition analysis was detected on the Thermo ICS 5000^+^ ion chromatography system (Thermo Fisher Scientific, Waltham, MA, USA) with Dionex™ CarboPac™ PA20 column (150 × 3.0 mm, 10 μm). The concentration of monosaccharide standards was 5 mg/mL. The injection volume was 5 μL. Mobile phases A, B and C were H_2_O, 0.1 M NaOH and 0.1 M NaOH with 0.2 M NaAc, and the flow rate was 0.5 mL/min. The column temperature was 30 °C [35].

#### 2.3.3. Molecular Weight (Mw) Analysis of PNRPs

The Mw analysis of PNRPs was conducted on high-performance gel permeation chromatography (HPGPC-ELSD) equipped with the Agilent 1260 Infinity ELSD evaporative light scattering detector, TSK-gel G-3000PWXL chromatographic column (7.8 × 300 nm) [36]. Chromatographic conditions: injection volume 10 μL. Distilled water was the mobile phase with a flow rate of 0.6 mL/min and a column temperature of 35 °C. Furthermore, 10 mg/mL of dextran standards with different molecular weights (Molecular Weight Standard from NIM) was used as the Mw standards.

#### 2.3.4. Fourier Transform-Infrared Spectroscopy (FT-IR) Analysis of PNRPs

PNRP powder and potassium bromide powder were pressed to perform the FT-IR analysis on a Nexus 470 FT-IR spectrometer (Equinx55, Bruce, Ettlingen, Baden-Württemberg, Germany). Samples were prepared by homogenizing 5 mg of dried polysaccharide with 500 mg of spectroscopic-grade KBr (1:100 *w*/*w*) in an agate mortar, followed by vacuum pressing at 10 MPa for 2 min to form translucent pellets. Spectra were acquired in absorbance mode over the range 4000–400 cm^−1^ at 4 cm^−1^ resolution with 32 scans per sample. Background correction was performed using a pure KBr pellet [37].

### 2.4. Characterization of Surface Morphology of PNRPs

PNRP powders were all sprayed with gold powder. A high-resolution field emission scanning electron microscopy system SU8010 (FE-SEM) (Hitachi, Tokyo, Japan) was applied to analyze the surface morphology of PNRPs under a voltage of 5.0 kV and secondary electron imaging (SEI) mode, with an image magnification of 1000 and 5000 times.

### 2.5. Chain Conformation Analysis of PNRPs

#### 2.5.1. Congo Red Examination

The helical structures of samples were investigated using the Congo red assay according to the method described [38]. First, 1 mL of each PNRPs’ solution (2 mg/mL) was mingled with 1 mL of Congo Red solution (200 μg/mL), and then different volumes of NaOH were added to make the concentration of NaOH from 0 to 0.5 M (diluted from a 5 M analytical-grade stock) with the final volume of 4 mL, and then reacted at room temperature for 5 min. Full wavelength scanning from 400 to 700 nm was recorded by a Microplate Reader (Tecan Spark, Männedorf, Switzerland), and the maximum absorption wavelength (λ_max_) was used for curve drawing.

#### 2.5.2. I_2_-KI Assay

Two mL of PNRPs (1 mg/mL) was mingled and reacted with 1.2 mL potassium iodide reagent (0.2% KI, and 0.02% I_2_, *w*/*v*) at room temperature for 10 min. Spectra were recorded by a Microplate Reader (Tecan Spark, Switzerland) in the range 300–700 nm [39].

### 2.6. Thermal Characteristic Identification

The thermal characteristics of PNRPs were determined by thermogravimetric analysis (TGA) and differential scanning calorimetry (DSC) on a Simultaneous Thermal Analyzer TGA/DSC1/1600LF (Mettler Toledo, Greifensee, Switzerland). For the TGA analysis, 4 mg of each PNRP powder in an aluminum crucible with a sealed gland was heated up from 40 °C to 800 °C at a rate of 10 °C/min with nitrogen at a flow rate of 40 mL/min. Furthermore, a similar condition was used for the DSC analysis, and only the changed condition of a flow rate of nitrogen was 50 mL/min [2].

### 2.7. Functional Characteristics analyse

#### 2.7.1. Water-Holding Capacity (WHC) and Oil-Holding Capacity (OHC)

WHC and OHC were determined according to the method of Abarghoei et al. [40]: 1000 mg of polysaccharides was added to 20 mL of distilled water or sunflower oil and homogenized at 4000 rpm for 30 s. The mixed samples were kept at room temperature for 30 min and then centrifuged. The centrifugation condition for WHC was 10,000× *g* for 25 min and 2000× *g* for 25 min for OHC. The unbound water or oil was separated. WHC and OHC were computed using the equations below:

(2) $$WHC=\frac{W_{2}-W_{1}}{W_{0}}$$

where W_0_ was the weight of the dry sample (g), W_1_ was the weight of the tube plus the dry sample (g) and W_2_ was the weight of the tube plus the sediment (g).

(3) $$OHC=\frac{O_{2}-O_{1}}{O_{0}}$$

where O_0_ was the weight of the dry sample (g), O_1_ was the weight of the tube plus the dry sample (g) and O_2_ was the weight of the tube plus the sediment (g).

#### 2.7.2. Emulsifying Activity Index (EAI) and Emulsifying Stability Index (ESI)

The evaluation of emulsifying characterstics was based on the improved method [41]. Aliquots (0.75 mL) of PNRPs’ dispersion (10 mg/mL) were mixed with soyabean oil (0.25 mL) and processed using an F6/10 Tissuelyser (Jingxin Company, Shaoxing, China) at 35,000 rpm for 60 s. Subsequently, 2 μL of the emulsion (0 and 10 min) was taken from the bottom and concentrated 100 times with 0.1% SDS to determine the absorbance at 500 nm using a Microplate Reader (Tecan Spark, Switzerland). The emulsification activity index (EAI) and emulsion stability index (ESI) of PNRPs were calculated using the following formula.

(4) $$EAI\;(m^{2}/g)=\frac{2\times 2.303\times A_{0}\times DF}{C\times \varphi \;\times 10000}$$

(5) $$ESI\;(\min)=\frac{A_{0}}{A_{0}-A_{10}}\times 10\;$$

A_0_ and A_10_ represent the absorbance of emulsions at 0 min and 10 min, respectively. DF denotes the dilution factor, which is 100 in this case. The variable C represents the concentration of polysaccharides in the samples, expressed in grams per milliliter (g/mL). Lastly, φ represents the oil bulk fraction, which is 0.25 in this context.

#### 2.7.3. Foaming Capacity (FC) and Foaming Stability (FS)

The measure of foaming peculiarities was conducted according to the previous method [41] with little adjustments. PNRPs’ dispersion (10 mg/mL) was mixed using a F6/10 Tissuelyser (Jingxin Company, China) at 35,000 rpm for 60 s. The foaming capacity (FC) and foam stability (FS) of PNRPs were determined using the formula:

(6) $$FC\;(\%)=\frac{V_{0}}{V}\;\times 100\;$$

(7) $$FS\;(\%)=\frac{V_{30}}{V}\times 100\;$$

where V (mL), V_0_ (mL) and V_30_ (mL) represent the original bulk of the dispersion, the bubble bulk at 0 min and 30 min, respectively.

### 2.8. In Vitro Antioxidant Abilities Analysis of PNRPs

The reducing power and scavenging abilities against ABTS, DPPH and hydroxyl radical of PNRPs were determined referred to the previous methods [42,43,44]. Ascorbic acid (Vc) was selected as the positive control. The antioxidant activity was further evaluated by determining the half inhibitory concentration (IC_50_).

### 2.9. In Vitro Immunomodulatory Activity of PNRPs

The in vitro immunomodulatory activity of PNRPs was evaluated by the method [45]. Lentinan and LPS were used as the positive controls. The specific method for cell viability measurement is as follows.

In total, 100 μL of RAW264.7 cells was inoculated at a density of 2 × 10^4^ cells/mL into 96-well plates overnight. Cells were treated with different concentrations of PNRPs (dissolved in the complete medium and formulated as 6.25, 12.5, 25, 50, 100 and 200 μg/mL) or LPS (2 μg/mL) for 24 h at 37 °C and 5% CO_2_, and each well was incubated with 10 μL of CCK-8 solution for 3 h at 37 °C. The absorbance was then measured at 450 nm determined by the Multifunctional Enzyme Labeling Instrument (Tecan Spark, Switzerland).

The effect of PNRPs on NO production was detected by the Griess method [46]. Furthermore, the levels of IL-6, IL-1β and TNF-α in the cell supernatant were determined using ELISA kits in accordance with the instructions of the ELISA kit.

### 2.10. Statistical Analysis

Data were analyzed by SPSS software (version 26.0 for Windows, IBM, Chicago, IL, USA). Differences among groups were conducted by a one-way analysis of variance (ANOVA) and Duncan’s multiple range test (*p* < 0.05). All experiments were conducted in triplicate.

## 3. Results and Discussion

### 3.1. Extraction Yield and Chemical Components of PNRPs

The yield and chemical components of PNRPs extracted by the six methods are listed in Table 1. Among the six extraction methods, PNRP-ABE had a lower extraction rate. In contrast, PNRP-HWE and PNRP-HAE had the highest extraction yields, which was consistent with previous reports that the HAE method produced the highest extraction yield of Pitaya Stem Powders (PSPs) compared with other different extraction methods [47]. The increase in extraction yield is due to physical mechanical damage, chemical reactions and the cell wall, while reducing the molecular weight of polysaccharides to increase the solubility of polysaccharides [48]. Alkali solutions disrupt polysaccharide ester/hydrogen bonds, inducing cellulose swelling to release intracellular polysaccharides [30]. The subsequent hydrolysis of the cell wall by hot alkaline solutions further enhances polysaccharide extraction and yield. The lower extraction yield of PNRP-AE is in line with the previous research that acid solution could destroy cell walls, and yet PSP belongs to acidic polysaccharides, which may be responsible for the low extraction yield [48]. Therefore, different extraction methods have a significant impact on the extraction yield of polysaccharides. Higher extraction yields do not inherently correlate with higher extraction yields of the target compound; a variety of other compounds may be extracted as well.

The polysaccharide content was above 75%. The protein content of PNRP-UAE was the lowest, while the protein content of PNRP-BE was higher. However, the protein content of PNRPs were all within the range of 0.14% to 0.19%. The total phenolic content of PNRPs ranged from 0.02 mg GAE/g to 0.03 mg GAE/g. The uronic acid content of PNRPs was from 5.17% to 7.67%. The results indicated that polysaccharides are the main bioactive substances in PNRPs.

#### 3.1.1. Monosaccharide Composition of PNRPs

**Figure 2:**
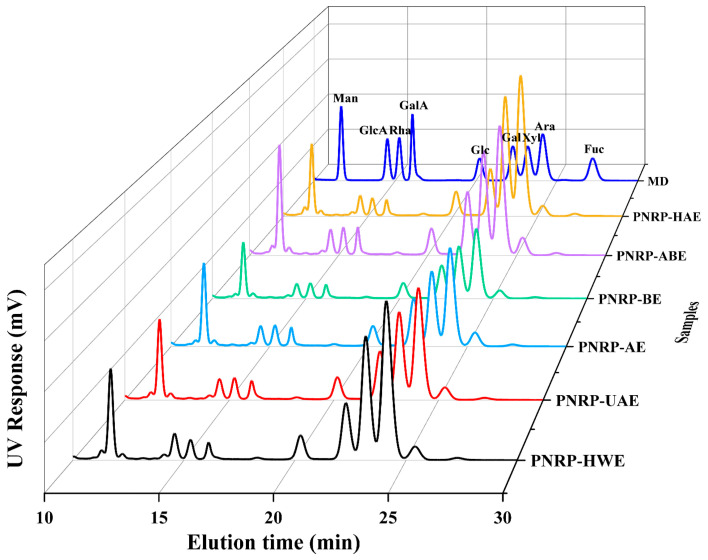
HPLC chromatograms of PNRPs. Monosaccharide standards (MD): mannose (Man), glucuronic acid (GlcA), rhamnose (Rha), galacturonic acid (GalA), glucose (Glc), galactose (Gal), xylose (Xyl), arabinose (Ara) and fucose (Fuc).

PNRPs were all composed of nine monosaccharides, containing Man, GlcA, Rha, GalA, Glc, Gal, Xyl, Ara and Fuc (Figure 2, Table S1). The types of main monosaccharides present in the six polysaccharides showed no significant differences across the extraction methods (*p* < 0.05). However, the relative molar ratios of these monosaccharides were significantly affected by the extraction method. *Pholiota nameko* polysaccharides only included five monosaccharides: Man (1.72%), Glu (14.27%), Gal (23.14%), Ara (50.32%) and Xyl (10.55%) [49]. The monosaccharide composition of *Pholiota nameko* polysaccharides and PNRPs was completely different. Moreover, the mole ratio of PNRPs was substantially changed, which was in line with alkali-extracted *Mung bean skin* polysaccharides (MBPs) [30]. The difference in the mole ratio of the monosaccharide composition of polysaccharides may be responsible for individual differences in their biological activities [50]. Research has shown that the higher content of galacturonic acid of polysaccharides leads to stronger antioxidant activity [51]. *Pholiota nameko* polysaccharides do not contain uronic acids, while PNRPs contain two types of uronic acids, accounting for 5.17 ± 0.08–7.67 ± 0.04%, which is consistent with the uronic acid content result in Table 1. The above results also suggest that the content of Xyl in PNRPs was the second highest compared with other monosaccharides. Xyl ranked the second highest monosaccharides, with PNRP-HAE containing the highest level (27.9%), a feature that may contribute to its strong antioxidant capacity. PNRP-AE possessed the highest content of Man (13.2%) and Gal (14.0%), and PNRP-BE contained the highest content of GalA (3.9%) and Glc (6.4%). In summary, PNRPs had the same type, but a different mole ratio of monosaccharides, which was in line with *Chrysanthemum indicum* L. polysaccharides [52].

#### 3.1.2. Mw of PNRPs

**Figure 3:**
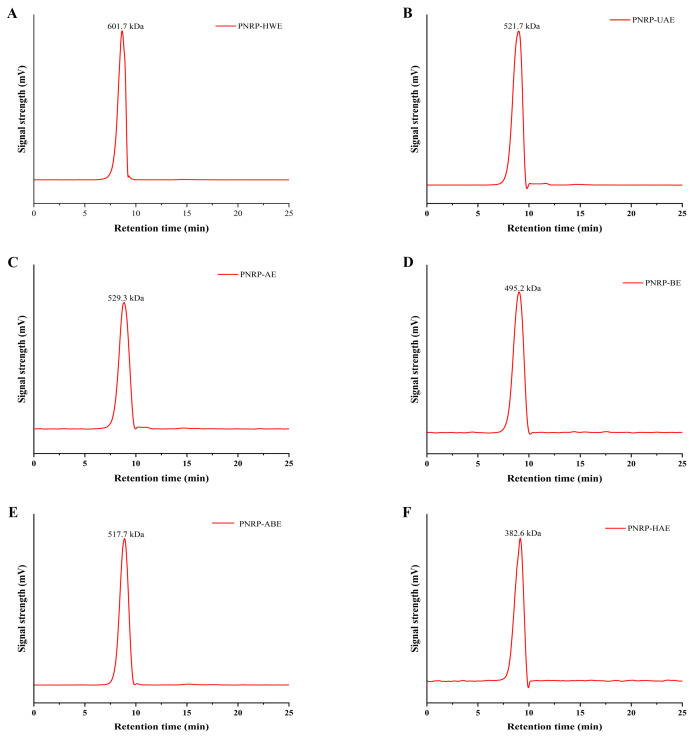
Molecular weight of PNRPs. (**A**) PNRP-HWE, (**B**) PNRP-UAE, (**C**) PNRP-AE, (**D**) PNRP-BE, (**E**) PNRP-ABE, (**F**) PNRP-HAE.

The Mw of the PNRPs from six extraction methods was determined by HP-GPC (Figure 3A–F). Extraction approaches had a remarkable impact on the Mw of PNRPs. The Mw of PNRP-HWE (601.7 kDa) was significantly larger than other PNRPs. The Mw of PNRP-UAE was 521.7 kDa, slightly lower than that of PNRP-HWE. Pu et al. [53] demonstrated that ultrasound treatment can decrease the molecular weight (Mw) of natural active polysaccharides [53]. The Mw of PNRP-AE was 529.3 kDa, which was lower than PNRP-HWE and PNRP-UAE, but higher than that of PNRP-BE, PNRP-ABE and PNRP-HAE. The HCl solution has a destructive effect on polysaccharides, leading to their degradation [54]. PNRP-HAE had the lowest molecular weight (382.6 kDa), significantly lower than other PNRPs. In accordance with the current results, high temperature and organic reagents can disrupt glycosidic bonds in polysaccharides, leading to a decline in Mw [55].

#### 3.1.3. FT-IR Analysis

**Figure 4:**
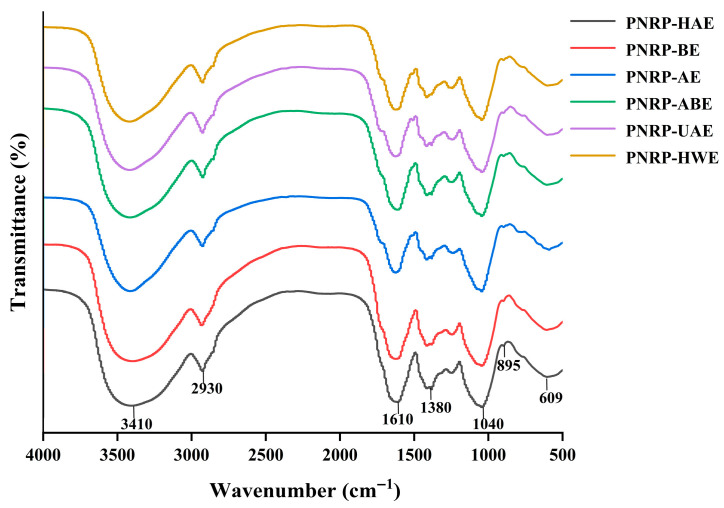
The FT-IR spectrum of PNRPs.

An FT-IR analysis is a common means of determining functional groups and glycosidic bond forms of polysaccharides. PNRPs possessed similar peaks (Figure 4), demonstrating that extraction methods did not influence the chemical functional groups of PNRPs. The wide and different characteristic peaks at 3600–3200 cm^−1^ were owing to the existence of OH^−^ in the polysaccharides, and the weaker peaks at 2900–2800 cm^−1^ were predominantly because of the asymmetrical stretching vibrations of C-H and -CH_2_ [56]. Characteristic peaks from 1700 to 1600 cm^−1^ were bound up with antisymmetric and symmetric C=O, implying the presence of uronic acid [57], which was in line with the content of galacturonic acid in PNRPs [58]. The peak at the range of 1200–1000 cm^−1^ could be caused by C-O-C stretching vibration, representing the possible occurrence of pyranose rings in polysaccharides [59]. The weak peak near 895 cm^−1^ implied the possible existence of the α-glycosidic linkage in the PNRPs [60]. Hence, PNRPs were novel polysaccharides possibly formed with pyranose rings and α-glycosidic bonds.

### 3.2. Surface Morphological Characterization

**Figure 5:**
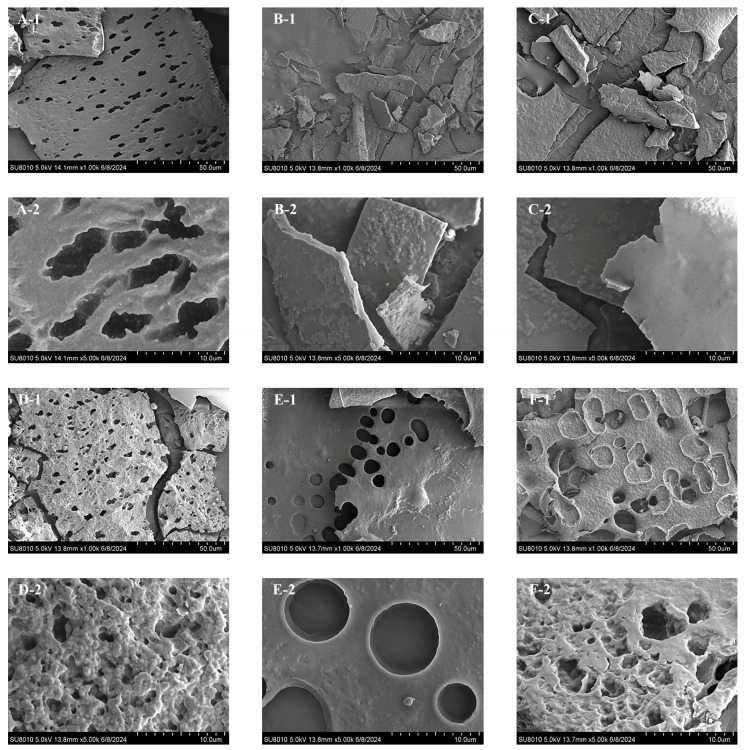
Surface morphology of PNRPs. (**A**) PNRP-HWE, (**B**) PNRP-UAE, (**C**) PNRP-AE, (**D**) PNRP-BE, (**E**) PNRP-ABE and (**F**) PNRP-HAE. (**-1**) 1000× and (**-2**) 5000×.

SEM is commonly employed for studying the surface morphological characteristics of polysaccharides, especially size, shape and porosity of macromolecules [61]. The surface morphological characteristics of PNRPs recorded by SEM are shown in Figure 5, which are significantly different. PNRP-HWE had a sheet-like structure with a porous structure and a fully folded paper-like structure, primarily owing to the fact that HWE maximizes the preservation of the original structure of polysaccharides, thereby enabling a better polymerization performance of polysaccharide chains and maintaining the integrity of longer molecular chains of polysaccharides [39]. PNRP-UAE exhibited a whole folded and integral paper-like shape with the smaller rough spherical structures, predominately because the ultrasound not only promoted the polysaccharides’ degradation but also rearranged polysaccharides. According to the results of Lv et al. (2024) [39], PNRP-AE had a non-regular layered structure and its surface was smoother compared to other extraction methods. PNRP-BE was in the form of paper sheets, and the magnified images showed circular or semi-circular pores with a diameter close to 10 µm on the surface. In addition, the connections between pores were cylindrical, indicating the corrosion by NaOH according to the previous results [62]. PNRP-ABE exhibited a porous sheet-like structure, and the resulting broken smooth sheets may be associated with the hydrolysis of polysaccharides by hydrochloric acid. PNRP-HAE showed loose sponge-like network structures, possibly caused by a mega-temperature plus alkaline conditions. The enlarged image shows a rough surface with irregular spherical particles, indicating a tight structure of PNRP-HAE. The surface morphology of plant polysaccharides is significantly influenced by extraction methods due to their varying effects on polysaccharide chain integrity; this has been demonstrated in polysaccharides from *Pyracantha fortuneana* [47] and *Fritillaria pallidiflora* Schrenk [63], where different approaches led to distinct degrees of hydrolysis or disruption and consequent morphological changes. The SEM results indicate that the different extraction methods dramatically impacted the surface morphology of PNRPs, which was similar with pumpkin polysaccharides [31].

### 3.3. Congo Red Assay

**Figure 6:**
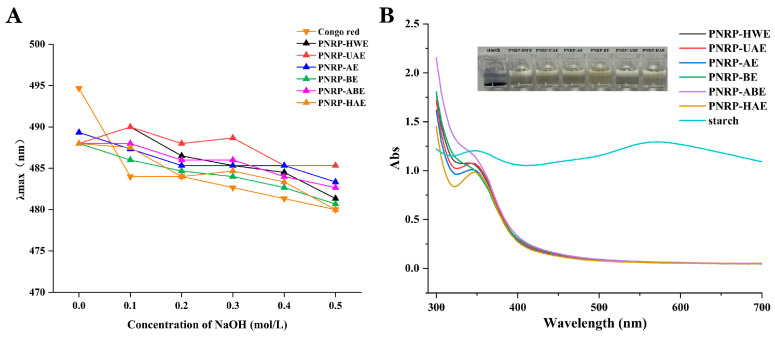
Congo red and I_2_-KI analysis of PNRPs. (**A**) The λ_max_ of Congo red PNRPs solution, (**B**) PNRPs in the presence of I_2_-KI.

The triple helix structure of polysaccharides is disrupted as the concentration of sodium hydroxide increases, and the λ_max_ shows a decreasing trend [64]. Hence, the Congo Red method was performed to detect the existence of triple helix structures (Figure 6A). The λ_max_ of the compound of six PNRPs showed a decreasing trend with an increasing concentration of sodium hydroxide, and no significant red shift or spin off phenomenon, illustrating the absence of a triple helix structure. Polysaccharides with a wide variety of monosaccharides do not easily form triple helix structures [65]. Hence, PNRPs possessed no triple helix structures.

### 3.4. I_2_-KI Analysis

An I_2_-KI analysis is commonly used to determine the existence of starch and long chains of polysaccharides. Polysaccharides do not exhibit a maximum absorption peak at 565 nm in an I_2_-KI solution, representing the presence of a complex molecular chain-like structure, longer molecular side chains and more massive branched chains [65]. The reaction solution showed no maximum absorption peak at 350 nm, indicating the presence of polysaccharides with intact and long branched chains [39]. PNRPs did not show a maximum absorption peak at 565 nm, implying that they were enriched with longer branched chains (Figure 6B). However, PNRP-HWE, PNRP-UAE, PNRP-AE and PNRP-HAE possessed maximum absorption peaks at 350 nm, whereas PNRP-BE and PNRP-ABE did not, implying that PNRPs extracted by HWE, UAE, AE and HAE contained more massive intact and long branched chains in comparison with those obtained by BE and ABE, which were similar to the *Pyracantha fortuneana* polysaccharides (PFPs) [39]. The solution after reaction still had the color of I_2_-KI and did not exhibit the characteristic color reaction of starch, illustrating that all six polysaccharides did not contain starch.

### 3.5. Thermal Stability Determination of PNRPs

**Figure 7:**
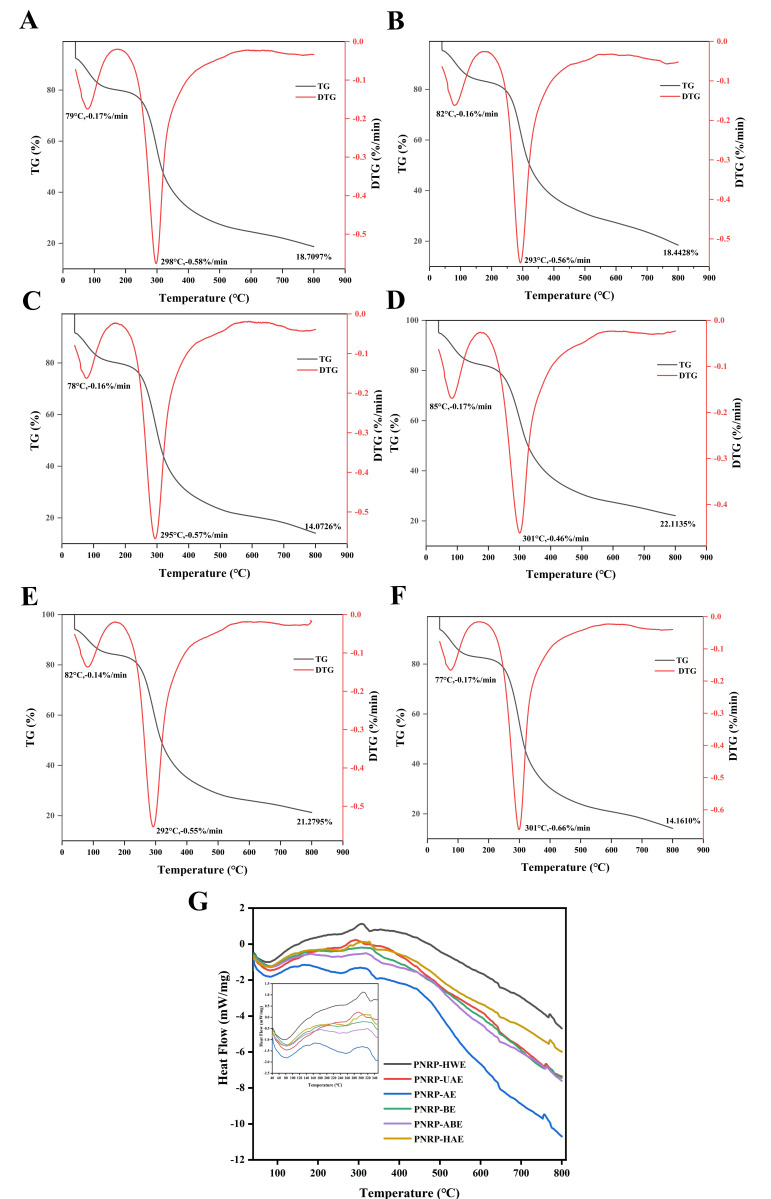
Thermal stability analysis of (**A**) PNRP-HWE, (**B**) PNRP-UAE, (**C**) PNRP-AE, (**D**) PNRP-BE, (**E**) PNRP-ABE, (**F**) PNRP-HAE and (**G**) the DSC curves of PNRPs.

Thermal stability is an important physicochemical property of polysaccharides to be used for industrial and commercial application [66]. The graphs of TG and DTG are shown in Figure 7A–F. Three processes of thermal weight loss appeared in PNRPs. Three thermal weight loss processes were observed in PNRPs. The initial process, occurring at 50–150 °C upon heating, corresponded to water loss in the polysaccharides and was observed in all six PNRP samples [67]. Then, a more rapid weight loss materialized between 100 °C and 240 °C, attributed to the structural depolymerization of polysaccharides. The existence of the second thermogravimetric process of PNRPs may be because of the existence of partially thermally unstable compounds in the polysaccharides [39]. Apparently, the weight loss of PNRP-BE was comparatively sluggish with the temperature rising, and the residual weight at 800 °C (22.11%) was larger than that of the other five PNRPs. The remaining weight of PNRP-AE was the lowest (14.07%). According to the DTG curves (Figure 7A,B), the maximum decomposition rates of PNRP-HWE, PNRP-UAE, PNRP-ABE and PNRP-ABE occurred at 298 °C, 293 °C, 295 °C and 292 °C, respectively. The maximum decomposition rates of PNRP-BE and PNRP-HAE both occurred at 301 °C. These results indicate that PNRP-BE and PNRP-HAE have better thermal stability than the other four PNRPs. The molecular weight of polysaccharides may be the reason for the difference in decomposition temperature [68].

The DSC curves further elucidated the thermal transitions of PNRPs with increased temperature (Figure 7G). The DSC curves of the preliminary PNRPs exhibited two endothermic peaks. The first endothermic peak transition was in the range of 40–100 °C, denoting the deprivation of free and bound water of PNRPs. The second endothermic peak transition was shown in PNRPs between 400 °C and 800 °C, meaning that PNRPs were broken down. Particularly, the heat flow of PNRP-AE was lower with increasing temperature, and lower than the other five PNRPs, similar to the TG result. The above data disclose that the distinct method significantly changed the thermal stability of PNRPs. The thermal stability of different PNRPs varied significantly, mainly due to the spatial structure changes induced by the extraction method, such as the dense network formed by the alkali treatment. In particular, the maximum decomposition temperature of PNRP-BE and PNRP-HAE was the highest (301 °C). The effect of molecular weight (Mw) on the thermal stability of polysaccharides was not uniform, depending on its ability to promote molecular alignment. How Mw affects thermal stability: While high molecular weight (HMW) may provide stronger intermolecular forces, chain entanglement tends to cause structural defects and amorphous regions, creating thermal degradation vulnerabilities [69]. Both PNRP-BE and PNRP-HAE treatments significantly reduced the molecular weight (Mw) of PNRPs. The decreased Mw facilitated more ordered molecular chains, enhanced crystallinity and substantially reduced chain entanglement and structural defects [70]. The alkali extraction method can significantly enhance the thermal stability of polysaccharides and is suitable for high-temperature processing applications [71]. Furthermore, the results indicate that PNRPs have high thermal stability and can be used to form biomaterials for various industrial applications.

### 3.6. Function Characteristics of PNRPs

#### 3.6.1. WHC and OHC

**Figure 8:**
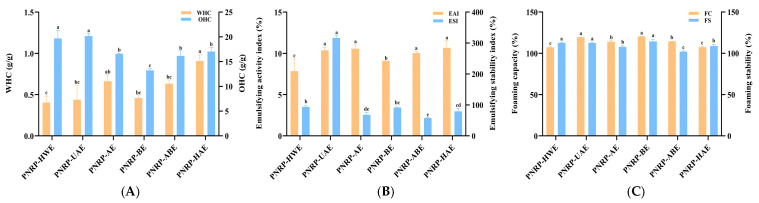
Functional properties of PNRPs. (**A**) WHC and OHC, (**B**) EAI and ESI, (**C**) FC and F. Different lowercase letters indicate significant differences between different groups (*p* < 0.05).

The functional characteristics of polysaccharides are linked to their chemical composition [72]. Among the significant functional properties of polysaccharides are WHC and OHC. Therefore, a sample with a higher WHC or OHC could be exploited and useful to hold onto water or oil, a preferred property of a good stabilizer in food [73]. Water-holding capacity (WHC) reflects the ability of food materials to retain water after undergoing different processing techniques. A higher WHC contributes to increased fecal output and bowel movement velocity, reducing rectal pressure and shortening intestinal transit time, thereby helping prevent gut-related disorders. Additionally, WHC can enhance satiety, supporting weight management. PNRP-HAE exhibited the highest WHC (0.9075 g/g), a finding consistent with SEM observations. The dense, mesh-like structure of PNRP-HAE facilitated water penetration into the fibers, enhancing its WHC. The WHC value of PNRP-HAE was comparable to that of FVP-MPs (81.72%), which was derived from *Flammulina velutipes* polysaccharide and porcine myofibrillar protein [74]. In contrast, PNRP-HWE, PNRP-UAE, PNRP-AE and PNRP-BE showed a lower WHC, likely due to their loose, porous structures. Overall, our results demonstrate that a porous microstructure reduced the WHC of PNRPs (Figure 8A). The WHC and OHC of PNRPs are closely related to their functional groups. Hydroxyl groups (-OH), abundant in PNRPs as indicated by the 3600–3200 cm^−1^ peak in FT-IR, enhanced WHC. They formed hydrogen bonds with water molecules, as seen in the study on steam-exploded apple pomace soluble dietary fiber [75], where increased -OH exposure boosted WHC. PNRPs with more accessible -OH groups, like PNRP-HAE with its dense structure, likely had stronger water binding.

Oil-holding capacity (OHC) reflects the ability of polysaccharides to bind oil, a property critical for food applications such as reducing excess dietary fat absorption [76], which is influenced by the hydrophilic–hydrophobic balance and surface characteristics of polysaccharides [77]. In this study, all PNRP exhibited notably high OHC values (Figure 8A). Additionally, PNRP-HWE and PNRP-UAE demonstrated the highest OHC (19.67 ± 1.59 g/g and 20.15 ± 0.46 g/g, respectively), likely because aqueous extraction preserved hydrophobic groups, facilitating oil penetration into its matrix [78]. In contrast, alkaline or acidic conditions disrupted the structural integrity of PNRPs, leading to reduced OHC. Certain hydrophobic functional groups contribute notably here: while most polysaccharide functional groups are hydrophilic, hydrophobic moieties like C-H and -CH_2_ (evidenced by the weak FT-IR peak at 2900–2800 cm^−1^) can interact with nonpolar oil molecules via van der Waals forces. In the current study, aqueous extracts (PNRP-HWE and PNRP-UAE), which likely preserved more hydrophobic structures, exhibited higher OHC than those obtained under alkaline or acidic conditions, as harsh extraction may disrupt hydrophobic domains and reduce OHC. PNRP-HWE and PNRP-UAE showed exceptional oil retention (19.67 ± 1.59 g/g and 20.15 ± 0.46 g/g, respectively), significantly exceeding the 13.39 g/g of Auricularia auricula glycoprotein (AAG) reported by Yang et al. [79]. This highlights the strong potential of PNRPs for food applications demanding high oil absorption/retention, such as fried food batters, high-fat fillings and fat substitutes.

#### 3.6.2. EAI and ESI

EAI and ESI are important indicators of emulsion functional properties, reflecting the ability of the complex to form an emulsified layer and its stability at the oil-in-water O/W interface [80]. As shown in Figure 8B, the EAI and ESI values of PNRP-UAE were 10.36 ± 0.36% and 316.26 ± 7.81%, respectively, indicating good emulsifying properties. PNRP-AE, PNRP-ABE and PNRP-HAE had EAI values of 10.55 ± 0.52%, 10.03 ± 0.19% and 10.66 ± 0.88%, respectively, exhibiting good emulsifying activity. Generally, many hydrophilic colloids or polysaccharides can serve as stabilizers in oil-in-water emulsions, but only a few act as emulsifiers. Emulsification properties require interfacial activity at the oil–water interface, enabling the formation and stabilization of fat particles during emulsion production. Polysaccharides are renowned for their water-retaining and thickening properties, which are attributed to their hydrophilic nature and high molecular weight. Therefore, they significantly influence the textural properties of food emulsions and extend their long-term shelf life. Research indicates that components with higher molecular weights exhibit lower stability [81]. PNRP-UAE demonstrated good emulsifying properties, which may be attributed to its lower molecular weight.

#### 3.6.3. FC and FS

The foaming capacity (FC) and foaming stability (FS) indices can comprehensively reflect the foam-related functional properties of polysaccharides in application scenarios such as food processing (e.g., as foaming agents, emulsifiers) and cosmetics (e.g., foam-based products) [82,83]. Owing to their abundant hydroxyl groups, polysaccharides exhibit pronounced hydrophilic properties, facilitating their retention in the aqueous phase of foam systems. This characteristic enhances the thickening and stabilizing properties of food products [84].

As illustrated in Figure 8C, the FC and FS values varied significantly across the tested samples. Notably, PNRP-UAE and PNRP-BE demonstrated superior foaming performance, with FC values of 119.33 ± 0.29% and 120.25 ± 0.35%, respectively, and comparable FS values, underscoring their exceptional ability to form and stabilize foams. These results align with the documented foaming properties of Chinese yam polysaccharides [85], suggesting their potential as effective functional ingredients in food applications.

In contrast, other extracts, such as PNRP-HWE, PNRP-AE, PNRP-ABE and PNRP-HAE, exhibited markedly lower FC and FS values (ranging from 50% to 100%), indicating comparatively weaker foaming capabilities. The pronounced performance of PNRP-UAE and PNRP-BE may be attributed to their structural characteristics resulting from their extraction methods. PNRP-UAE’s homogeneous mid-weight flexible chains facilitated rapid interfacial adsorption, while PNRP-BE’s long-chain hydrogen-bond networks enhanced film stability which enhanced interfacial adsorption and film formation. These findings highlight the importance of extraction methods in modulating the functional properties of polysaccharides.

### 3.7. In Vitro Antioxidant Abilities of PNRPs

**Figure 9:**
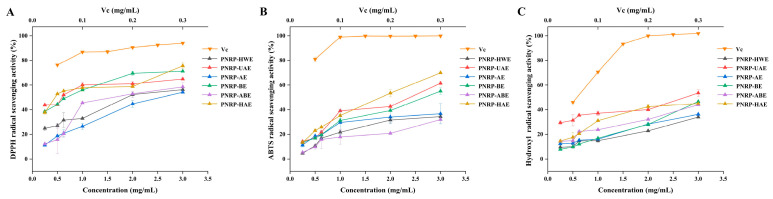
In vitro antioxidant abilities of PNRPs. (**A**) DPPH radical scavenging ability, (**B**) ABTS radical scavenging ability, (**C**) hydroxyl radical scavenging ability.

The DPPH scavenging abilities of PNRPs were dose-dependently increased (Figure 9A). The clearance rates against DPPH radicals of PNRP-HWE, PNRP-UAE, PNRP-AE, PNRP-BE, PNRP-ABE and PNRP-HAE at 3 mg/mL were 56.29 ± 0.26%, 64.97 ± 0.94%, 54.20 ± 0.37%, 71.26 ± 0.23%, 58.54 ± 3.57% and 75.59 ± 2.34%, respectively. PNRP-HAE, PNRP-UAE and PNRP-BE had a similar ability to scavenge DPPH radicals, with maximum scavenging rates exceeding 60%, but all lower than Vc. The maximum clearance rates of PNRP-ABE, PNRP-HWE and PNRP-AE were all above 50%. The IC_50_ values on the DPPH radical scavenging ability of PNRPs are listed in Table 2. PNRP-BE (0.57 mg/mL), PNRP-UAE (0.58 mg/mL) and PNRP-HAE (0.59 mg/mL) had lower values of IC_50_. Additionally, PNRP-HAE had the highest xylose content (27.9%), which may contribute to its high antioxidant activity. Consistent with findings on *Flammulina velutipes* residue by an ultrasonic-assisted snailase enzymatic extraction method (UE-FVRP) [86], this 27.9% of Xyl content in PNRP-HAE positively correlated with its excellent DPPH scavenging rate (86.7% at 2 mg/mL), confirming a structure–activity relationship.

The scavenging activity of PNRPs on ABTS radicals was analogue to that of DPPH radicals (Figure 9B). PNRP-HAE (69.97 ± 0.53%), PNRP-UAE (61.49 ± 1.40%) and PNRP-BE (55.09 ± 2.64%) with 4 mg/mL had higher scavenging rates, exceeding 55%, but were all lower than that of Vc (99.95 ± 0.06%). The maximum clearance rates of PNRP-ABE (32.07 ± 0.47%), PNRP-HWE (34.49 ± 1.28%) and PNRP-AE (36.74 ± 4.41%) were all above 30%. Lower IC_50_ was exhibited in PNRP-HAE (1.57 mg/mL), PNRP-UAE (2.00 mg/mL) and PNRP-BE (3.42 mg/mL) (Table 2).

PNRPs dose-dependently increased in the scavenging ability on OH^−^ radicals (Figure 9C). PNRP-UAE (53.59 ± 2.61%), PNRP-BE (46.64 ± 0.33%) and PNRP-HAE (44.99 ± 0.36%) exhibited relatively high scavenging rates (40%) at 4 mg/mL, but still lower than that of Vc (101.91 ± 0.15%). The maximum clearance rates of PNRP-ABE, PNRP-AE and PNRP-HWE were all above 30%. PNRP-UAE (3.00 mg/mL), PNRP-BE (3.72 mg/mL) and PNRP-HAE (3.97 mg/mL) had lower values of IC_50_ (Table 2).

On the basis of the above-mentioned results, PNRP-HAE, PNRP-UAE and PNRP-BE had the highest antioxidant activity. The molecular weight of polysaccharides and the antioxidant activities exhibited a negative relationship, possibly due to the fact that a higher molecular weight limits the diffusivity of polysaccharides and the interaction with free radicals, while a lower molecular weight allows more hydroxyl terminals to be reduced [87]. Hence, the higher antioxidant activity of PNRP-HAE and PNRP-BE may be due to their smaller molecular weight. Additionally, the molecular weight of PNRP-HAE was lower than that of PNRP-BE. However, the antioxidant activity of PNRP-HAE was lower than that of PNRP-BE, probably related to the high ratio of galacturonic acid and low molecular weight of PNRP-HAE [88]. The antioxidant activities of polysaccharides are not influenced by a single factor, but multiple factors, comprising monosaccharide composition, molecular weight, glycosidic bond type and chain conformation [89]. PNRP-UAE with higher Mw had high antioxidant abilities, which may be attributed to its triple helix structure, richer branched chains and higher gel property [90]. This result was also analogue to the results of *Pyracantha fortuneana* polysaccharides [39]. The contents of Rha, Glu and GalA in monosaccharides are associated with antioxidant activity [91]. In the current study, PNRP-HAE had the strongest antioxidant capacity, which had the smallest molecular weight and the lowest molar ratio of Rha (2.9%), Glu (6.1%) and GalA (10.8%). PNRP-AE had a lower antioxidant capacity with a larger molecular weight, and the highest molar ratio of Rha (4.2%), Glu (6.3%) and GalA (14.0%). This result is consistent with *Astragalus* polysaccharides [25].

### 3.8. In Vitro Immunoregulatory Activity of PNRPs

#### 3.8.1. The Effect of PNRPs on the Viability of RAW264.7 Cells

**Figure 10:**
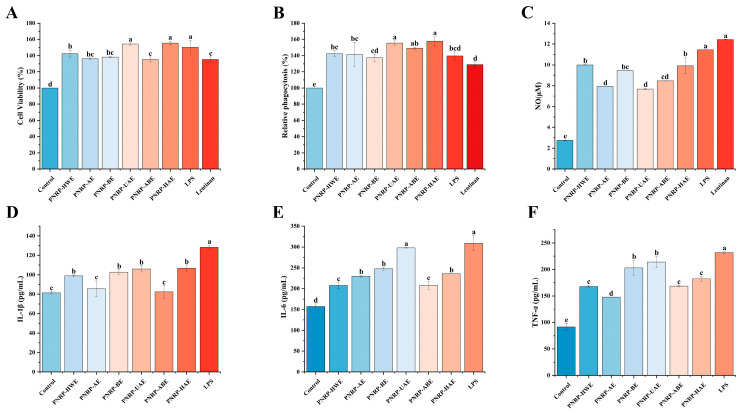
In vitro immunoregulatory activity of PNRPs. (**A**) Cell viability, (**B**) relative phagocytic, (**C**) NO, (**D**) IL-1β, (**E**) IL-6 and (**F**) TNF-α by RAW264.7 cells. Control: normal cells. Different lowercase letters indicate significant differences between different groups (*p* < 0.05).

RAW264.7 cells, widely distributed in the immune system, play a crucial role in nonspecific immunity [92]. The cytotoxic effects of polysaccharides were evaluated to assess the impact of six polysaccharides (PNRP-HWE, PNRP-UAE, PNRP-AE, PNRP-BE, PNRP-ABE, PNRP-HAE) on cell viability. Compared to the control group, 200 μg/mL PNRPs exhibited no toxicity and significantly enhanced cell viability, with PNRP-HAE and PNRP-UAE demonstrating the strongest activity (Figure 10A). These findings indicate that at the tested concentrations, PNRP-HWE, PNRP-UAE, PNRP-AE, PNRP-BE, PNRP-ABE, PNRP-HAE, Lentinan and LPS showed no cytotoxicity toward RAW264.7 cells. Ge et al. demonstrated that isochlorogenic acid A at 10–400 μg/mL increased RAW264.7 cell viability, higher than the blank group but lower than the LPS, thereby indicating its role in stimulating the proliferation of RAW264.7 cells [93]. The proliferation of RAW264.7 cells post-treatment is a well-recognized marker of immune cell activation, as activated macrophages expand their effector population via proliferation to augment phagocytosis, cytokine production and antigen presentation—key processes in innate and cell-mediated immunity [94]. Lentinan is a type of polysaccharide with immunomodulatory effects [95]. PNRPs (200 μg/mL) had similar effects in proliferation ability to those of LPS and Lentinan, indicating that PNRPs showed excellent macrophage activation ability. This congruence in proliferative activity further validates RAW264.7 cell viability/proliferation as a robust metric for assessing immune-enhancing potential [96]. Collectively, PNRPs, particularly PNRP-HAE and PNRP-UAE, function as potent macrophage activators. The significant enhancement in cell viability provides compelling evidence for their immunostimulatory activity.

#### 3.8.2. The Effect of PNRPs on the Phagocytic Ability of RAW 264.7 Cells

Phagocytosis is a significant barrier for the host to enhance the innate immunomodulatory system [45]. As shown in Figure 10B, RAW264.7 macrophages stimulated with PNRPs exhibited significantly greater phagocytic activity than the control group, indicating all PNRPs had the ability to promote phagocytic activity. Lentinan enhances antitumor immune responses by activating immune cells (such as NK cells, T cells and dendritic cells) and can act as a biological response modifier (BRM) to improve the efficacy of chemotherapy while reducing immunosuppression and toxicity. Its immunomodulatory mechanisms include promoting cytokine secretion (such as IL-2 and IFN-γ) and improving the immune function of cancer patients, making it a potential adjuvant drug for chemotherapy [97]. Except PNRP-BE, the PNRPs showed a higher effect on promoting the phagocytic activity of RAW264.7 cells than that of Lentinan at the same concentration (*p* < 0.05). Furthermore, PNRP-UAE and PNRP-HAE even resulted in the highest phagocytic capacity, reaching 1.55-fold and 1.57-fold more than that of Lentinan, respectively. Therefore, PNRP-UAE and PNRP-HAE were expected to be more effective than Lentinan. The immunomodulatory activity of polysaccharides is closely related to their structure, and molecular weight is a key factor influencing their interaction with receptors [98]. For instance, Deng et al. explored interactions between *Lycium barbarum* polysaccharides (LBP) of varying molecular weights and the TLR4/MD-2 complex, finding medium-molecular-weight LBP showed a higher affinity for TLR4-related receptors, and fractions <350 kDa were key active components enhancing macrophage function [99]. In contrast, high-molecular-weight polysaccharides formed entangled macromolecular aggregates in the solution, increasing steric hindrance, fixing molecular conformations, enhancing rigidity and reducing receptor-binding efficiency due to their hindered close contact from rigid backbones and aggregation [100]. This significant enhancement suggests that the structural features of PNRP-UAE and PNRP-HAE (such as glycosidic bonds and molecular weight) may optimize receptor binding on immune cells.

#### 3.8.3. The Effect of PNRPs on the Secretion of NO and Cytokines in RAW264.7 Cells

Activated macrophages secrete substantial amounts of secondary metabolites, including NO, IL-1β, IL-6 and TNF-α [101]. NO serves as a fundamental signaling molecule involved in key cellular functions, and its overproduction by activated phagocytes is critical for pathogenelimination [102]. IL-1β can activate immune cells, assist in T cell proliferation, participate in antibody production and promote inflammation [103]. IL-6, a pleiotropic soluble mediator, plays vital roles in inflammation, immune responses and hematopoiesis. It bridges innate and adaptive immunity by promoting the specific differentiation of naïve CD4^+^ T cells [103]. TNF-α not only activates macrophages but also enhances their phagocytic capacity [104]. Thus, these molecules (NO, IL-1β, TNF-α and IL-6) serve as reliable indicators for evaluating the immunomodulatory potential of natural compounds.

As illustrated in Figure 10C, PNRP-UAE and PNRP-HAE significantly enhanced NO secretion in RAW264.7 cells compared to the control. At 200 μg/mL, NO production peaked at 11.53 μM (5.19-fold higher than the control), though it remained lower than the LPS group. As shown in Figure 10D, PNRP-HWE, PNRP-BE, PNRP-UAE and PNRP-HAE significantly enhanced IL-1β secretion in RAW264.7 cells compared to the control group, although the levels remained lower than those in the LPS-treated group. Figure 10E demonstrates that all six polysaccharides markedly increased IL-6 secretion. At 200 μg/mL, PNRP-HWE, PNRP-AE, PNRP-BE and PNRP-UAE exhibited dose-dependent enhancement, with PNRP-UAE achieving maximal IL-6 secretion (298.125 pg/mL), comparable to the LPS-positive control. Similarly, Figure 10F reveals that all polysaccharides significantly upregulated TNF-α secretion across the tested concentrations. At 200 μg/mL, TNF-α levels induced by all six polysaccharides surpassed the control, with PNRP-UAE and PNRP-BE yielding the highest concentrations (albeit lower than LPS). Collectively, these results highlight PNRP-UAE and PNRP-HAE as the most potent immunomodulatory agents. PNRP-HAE and PNRP-UAE represent a unique class of plant-derived immunomodulators, each exhibiting distinct and complementary functional advantages. Both PNRP-UAE and PNRP-HAE demonstrate strong capabilities in enhancing cellular vitality, phagocytic activity and IL-1β secretion. PNRP-HAE exhibits superior efficacy in enhancing nitric oxide (NO) production, while PNRP-UAE strongly stimulates the secretion of key immune modulatory factors (IL-6 and TNF-α). Notably, both polysaccharides outperform Lentinan in core functional metrics while maintaining excellent biocompatibility. Future research should prioritize structure-driven product development and rigorous clinical validation to fully unlock their potential as next-generation vaccine adjuvants, antimicrobial potentiators or functional food components.

## 4. Conclusions

This study systematically compared six extraction methods for isolating polysaccharides from *Pholiota nameko* residue (PNRPs), demonstrating that extraction techniques significantly influence extraction yield, structural properties and bioactivities. PNRP-HAE emerged as the most promising candidate due to its high extraction yield, low molecular weight and exceptional antioxidant and immunomodulatory capacities. Structural characterization revealed that PNRPs share core features (e.g., α-glycosidic bonds, triple helix conformation), but differ in monosaccharide composition and thermal stability, with PNRP-HAE and PNRP-BE showing enhanced thermal resistance. The strong bioactivities of PNRP-HAE and PNRP-UAE, particularly in scavenging free radicals and activating immune responses, underscore their potential as functional food ingredients. These results provide a scientific foundation for optimizing PNRP extraction and advancing the utilization of mushroom residue in health-promoting applications.

## Figures and Tables

**Table 1:** The extraction yield and chemical compositions of PNRPs.

	Extraction Yield (%, *w*/*w*)	Polysaccharides (%, *w*/*w*)	Total Uronic Acids (%, *w*/*w*)	Total Proteins (%, *w*/*w*)	Total Phenols (mg GAE/g)
PNRP-HWE	4.24 ± 0.22 ^a^	92.56 ± 1.17 ^a^	5.17 ± 0.08 ^f^	0.18 ± 0.004 ^b^	0.03 ± 0.0002 ^a^
PNRP-UAE	3.08 ± 0.53 ^b^	90.33 ± 0.09 ^b^	7.67 ± 0.04 ^a^	0.14 ± 0.006 ^e^	0.03 ± 0.0001 ^a^
PNRP-AE	3.01 ± 0.43 ^b^	75.68 ± 0.12 ^f^	7.43 ± 0.06 ^b^	0.17 ± 0.003 ^c^	0.03 ± 0.0001 ^a^
PNRP-BE	2.57 ± 0.67 ^b^	77.89 ± 0.78 ^e^	5.37 ± 0.01 ^e^	0.19 ± 0.001 ^a^	0.02 ± 0.0001 ^b^
PNRP-ABE	1.09 ± 0.22 ^c^	81.54 ± 0.05 ^d^	6.66 ± 0.11 ^c^	0.15 ± 0.001 ^d^	0.02 ± 0.0001 ^b^
PNRP-HAE	3.92 ± 0.24 ^a^	85.12 ± 0.05 ^c^	6.06 ± 0.14 ^d^	0.18 ± 0.002 ^b^	0.03 ± 0.0001 ^a^

Different lowercase means the significance among different groups (*p* < 0.05).

**Table 2:** IC_50_ value for in vitro antioxidant activity of PNRPs.

Variety	IC_50_ Values for DPPH (mg/mL)	IC_50_ Values for ABTS (mg/mL)	IC_50_ Values for Hydroxyl (mg/mL)
Vc	0.07 ± 0.0075 ^d^	0.05 ± 0.0003 ^e^	0.17 ± 0.0013 ^f^
PNRP-HWE	1.91 ± 0.0695 ^b^	5.86 ± 0.1160 ^ab^	8.37 ± 0.3410 ^a^
PNRP-UAE	0.58 ± 0.0202 ^c^	2.00 ± 0.0505 ^d^	3.00 ± 0.1920 ^e^
PNRP-AE	2.60 ± 0.0735 ^a^	5.20 ± 0.8065 ^b^	6.71 ± 0.0980 ^b^
PNRP-BE	0.57 ± 0.0015 ^c^	3.42 ± 0.0295 ^c^	3.72 ± 0.0710 ^d^
PNRP-ABE	1.89 ± 0.1590 ^b^	7.12 ± 0.3180 ^a^	5.55 ± 0.0655 ^c^
PNRP-HAE	0.59 ± 0.0164 ^c^	1.57 ± 0.0290 ^d^	3.97 ± 0.1145 ^d^

Different lowercase means significant differences between groups (*p* < 0.05).

## Data Availability

The original contributions presented in this study are included in the article/Supplementary Materials. Further inquiries can be directed to the corresponding authors.

## Supplementary Materials

The following supporting information can be downloaded at: https://www.mdpi.com/article/10.3390/foods14173071/s1, Table S1: Monosaccharide composition of PNRPs.
