# Supplementary material for: Impacts of Six Methods of Extraction on Physicochemical Properties, Structural Characteristics and Bioactivities of Polysaccharides from Pholiota nameko Residue
Source: Foods. 2025 Aug 30;14(17):3071. doi: 10.3390/foods14173071 (PMC12427657; doi:10.3390/foods14173071)
Supplement: Supplementary file 1 [file foods-14-03071-s001.zip › foods-3766415-supplementary.pdf]

Single sugar composition analysis empirical method:

(1) Acid hydrolysis:

1) Weigh 1mg of the sample in a small bottle for acid hydrolysis, add 1ml of methanol hydrochloric acid solution, and react with N2 at 80°C under a constant temperature metal bath for 16h.

2) After the reaction, the methanol hydrochloric acid in the sample was dried by nitrogen blowing instrument, and 1ml of 2M trifluoroacetic acid (TFA) was added to the reaction for 1h at 120°C. After the reaction, it was dried again.

(2) Derivatization of monosaccharides:

1) Add 500ul of 0.3M NaOH to the acid hydrolysis vial to completely dissolve the dry monosaccharide sample.

2) Add 500ul 0.5M PMP-methanol, and it can be seen that PMP and NaOH rapidly diffuse and mix together, and the insoluble material at the bottom is dispersed uniformly by blowing with a gun.

3) Take 200ul of the mixture into the EP tube, and put the sample into a 70°C water bath for 30min.

4) Take out the reaction sample and add 100ul 0.3M HCL. After adding HCL, a large amount of precipitate will appear, followed by extraction.

5) Add 700ul dichloromethane, and the organic phase and water phase will be clearly separated.

6) Vortex the EP tube for 90s until completely mixed, then centrifuge. At this time, the organic phase and water phase are completely separated. Use a flat-tipped syringe to

pump out the lower organic phase.

7) Repeat step 6 twice.

8) The remaining aqueous phase was aspirated with a 1ml syringe and filtered through a 0.22um organic filter membrane, and then sampled in HPLC.

Table S1. Monosaccharide composition of PNRPs.

	Man	GlcA	Rha	GalA	Glc	Gal	Xyl	Ara	Fuc
**PNRP-HWE**	10.6 %	3.7 %	2.8 %	2.5 %	4.6 %	11.6 %	26.4 %	37.3 %	0.6 %
**PNRP-UAE**	12.0 %	3.8 %	3.9 %	3.6 %	5.5 %	12.8 %	24.3 %	33.4 %	0.7 %
**PNRP-AE**	13.2 %	4.1 %	4.2 %	3.9 %	6.3 %	14.0 %	22.4 %	31.2 %	0.7 %
**PNRP-BE**	12.7 %	4.2 %	4.4 %	4.5 %	6.4 %	13.7 %	22.1 %	31.3 %	0.8 %
**PNRP-ABE**	13.0 %	3.9 %	4.1 %	4.2 %	6.3 %	13.7 %	23.0 %	31.1 %	0.8 %
**PNRP-HAE**	9.1 %	3.2 %	2.9 %	2.9 %	6.1 %	10.8 %	27.9 %	36.3 %	0.7 %

Man: mannose, GlcA: glucuronic acid, Rha: rhamnose, GalA: galacturonic acid, Glc: glucose, Glc: glucose, Gal: galactose, Xly: xylose, Ara: arabinose，Fuc: fucose.
